# Free Osteocutaneous Fibular Flap for Hand Reconstruction Related to Clear Cell Sarcoma Resection: A Case Report

**DOI:** 10.7759/cureus.113237

**Published:** 2026-07-23

**Authors:** Cesar A Gonzalez-Martinez, Jorge A Gutierrez-Gonzalez, Daniel Salas-Trevino, Cynthia M González-Cantú, Yanko Castro-Govea

**Affiliations:** 1 Plastic and Reconstructive Surgery, University Hospital "Dr. Jose Eleuterio Gonzalez" Autonomous University of Nuevo Leon, Monterrey, MEX; 2 Infectious Diseases, University Hospital "Dr. Jose Eleuterio Gonzalez" Autonomous University of Nuevo Leon, Monterrey, MEX

**Keywords:** clear cell sarcoma, free fibula flap, hand and microsurgery, hand reconstruction, soft tissue sarcoma

## Abstract

We present a 25-year-old female with a two-year history of clear cell sarcoma (CCS) of the hand. The patient presented with a painless left hypothenar tumor without neurological or functional compromise. MRI was compatible with sarcoma (4.8x3.4x4.9cm) and incisional biopsy confirmed CCS by immunohistochemistry (MITF1 and EWSR1 rearrangement both positive). The patient received preoperative radiotherapy for tumor reduction (approximately 80%). A wide resection was performed with negative margins of 2 cm, including third-fifth metacarpals, partial carpectomy and amputation of the fifth finger due to intraoperative ischemia. The lesion was reconstructed with a free fibula flap whose survival and postoperative short-term evolution were favorable. Six months after surgery, the patient presented partial mobility under rehabilitation with no evidence of recurrence or metastasis. Disabilities of the Arm, Shoulder and Hand (DASH), Musculoskeletal Tumor Society (MSTS) score, grip and pressure strength could not be determined.

Our case is very particular due to the hypothenar involvement with central multistructural involvement and an atypical slow evolution. The aggressive therapeutic approach with microsurgical resection, reconstruction and functional preservation using a multisegmented fibula free osteocutaneous flap was favorable for the patient’s outcome.

## Introduction

Clear cell sarcoma (CCS) is a soft tissue sarcoma (STS) that arises from the tendon and aponeurosis [1-3]. Described by Enzinger in 1965 [4], it accounts for less than 1% of all STSs, with an estimated incidence of 0.014 per 100,000 people [1,3,5]. It stands out for its high metastatic spread and a poor prognosis [6].

In the approach to a tumor, radiography helps to discern bone or soft components. Ultrasound (US) can report cystic or solid lesions, computed tomography (CT) details bone characteristics, and magnetic resonance imaging (MRI), the method of choice to detail soft tissue lesions, informs the relationship with neighboring structures [2,5,7]. 

Diagnostic confirmation requires biopsy, preferably by percutaneous technique with an image-guided core needle, or, failing that, an open biopsy can be used. This approach is essential to define the therapeutic strategy and establish the prognosis [2,8]. Treatment includes en bloc resection; hand amputation is the most common outcome for this sarcoma due to its aggressiveness. Adjuvant radiation therapy is described in lesions >5 cm [2].

In the present report, the lesion resulting from the resection of the CCS showed significant structural alterations in the patient's hand. Therefore, the plastic surgery team performed a reconstruction technique specifically planned to maximize the functional restoration of the affected hand.

## Case presentation

In this study, we present the diagnostic and therapeutic approach of a 25-year-old female patient with a two-year evolution of a CCS in the hand. No comorbidities or family history of STSs were reported. The disease began two years earlier with increased left hypothenar volume, accompanied by mild pain.

On targeted examination, the left hand showed an increase in hypothenar volume, and on palpation, a tumor of approximately 5x3x4 centimeters (cm), soft, not very mobile, not painful to manipulation and without changes in coloration. No functional or neurosensory limitations.

**Figure 1:**
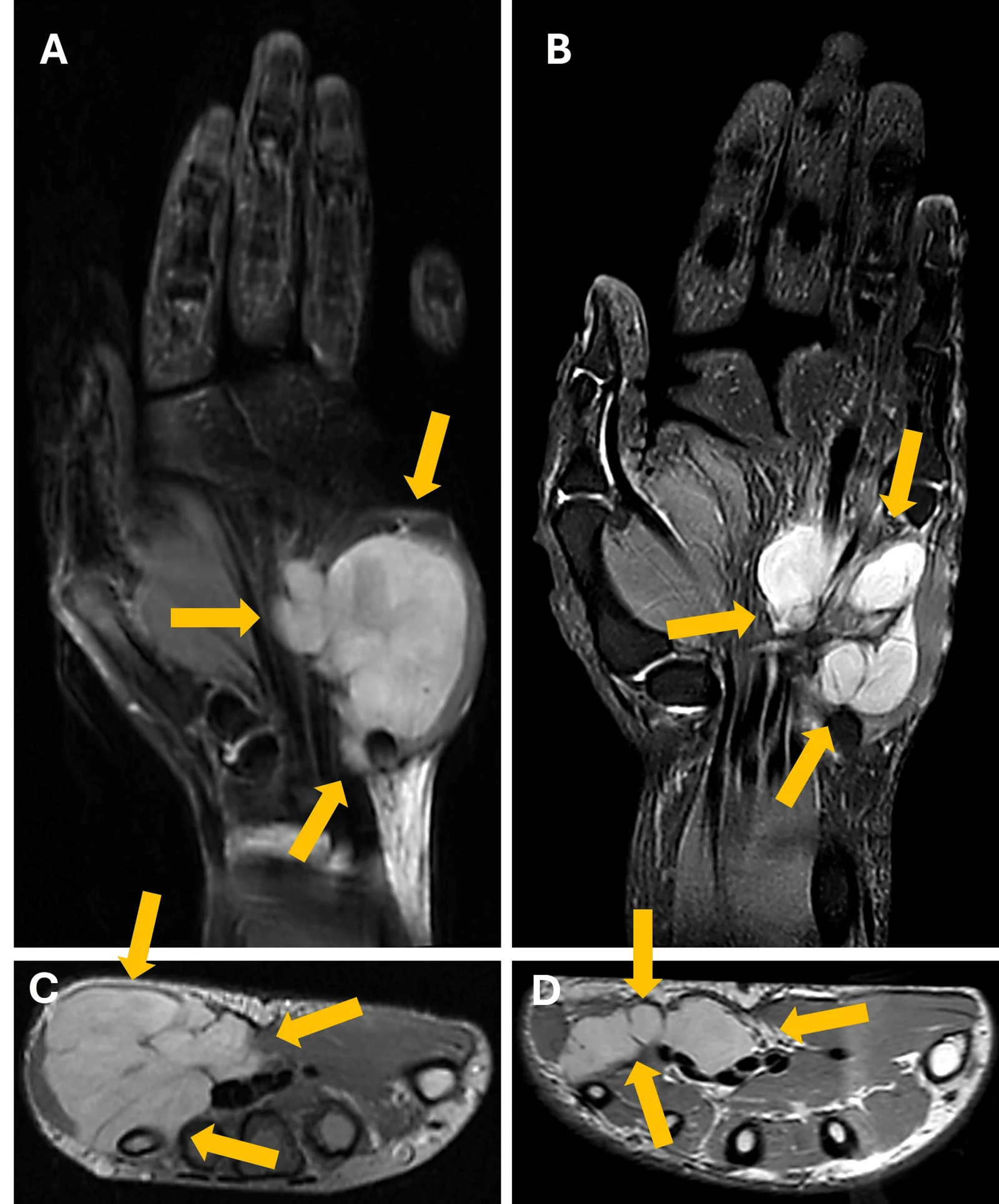
Magnetic resonance imaging of the clear cell sarcoma of the hand Pre-radiotherapy: Multilobulated, predominantly cystic tumor in the hypothenar eminence, with thin hypointense septa, measuring 4.8 x 3.4 x 4.9 cm, extending into the space between the carpal tunnel and the hamate bone in the lateral palmar septum, encompassing the fourth and fifth metacarpals (MTC) in A) coronal and C) axial view. Post-radiotherapy: Tumor with the same characteristics but smaller, measuring 3 x 3.1 x 3.7 cm (previously 1.2 x 3.1 x 4.5 cm), displacing the carpal and cubital tunnel anteriorly, in B) coronal view and D) axial view. The margins of the tumor are marked with orange arrows.

MRI showed findings suggestive of STS, with a solid-appearing, multilobulated lesion observed at the hypothenar eminence, without calcifications, containing hypointense septa (Figure 1A, 1C). It appeared hyperintense on fat-suppressed and gradient-echo sequences, with areas of restricted diffusion and anti-diffusion contrast (ADC) and heterogeneous enhancement after gadolinium administration. The lesion measured approximately 4.8 x 3.4 x 4.9 cm along its anteroposterior, laterolateral, and craniocaudal axes, with an approximate volume of 41 cc. It extended inferiorly into the space between the carpal tunnel and the hamate bone in the lateral palmar septum, anteriorly into the interosseous space encompassing the metacarpals (MTCs) of the fifth and fourth phalanges, and medially anterior to the flexor retinaculum. This lesion caused slight compression and contralateral deviation of the carpal tunnel, predominantly affecting the deep flexor tendons of the fifth finger. These findings, taken together, are suggestive of synovial sarcoma. Based on this, an incisional biopsy (for economic limitations) was taken, and the histopathological report concluded CCS.

Subsequently, the patient received 25 sessions of 50 Gy external-beam radiotherapy, after which the lesion measured approximately 1.3 x 3.1 x 3.7 cm with an approximate volume of 2 cc. It showed no enhancement after gadolinium administration. The lesion displaced the carpal and cubital tunnels anteriorly without involvement of tendons, muscles, bones, or vascular structures, as evidenced by control MRI (Figures 1B, 1D).

**Figure 2:**
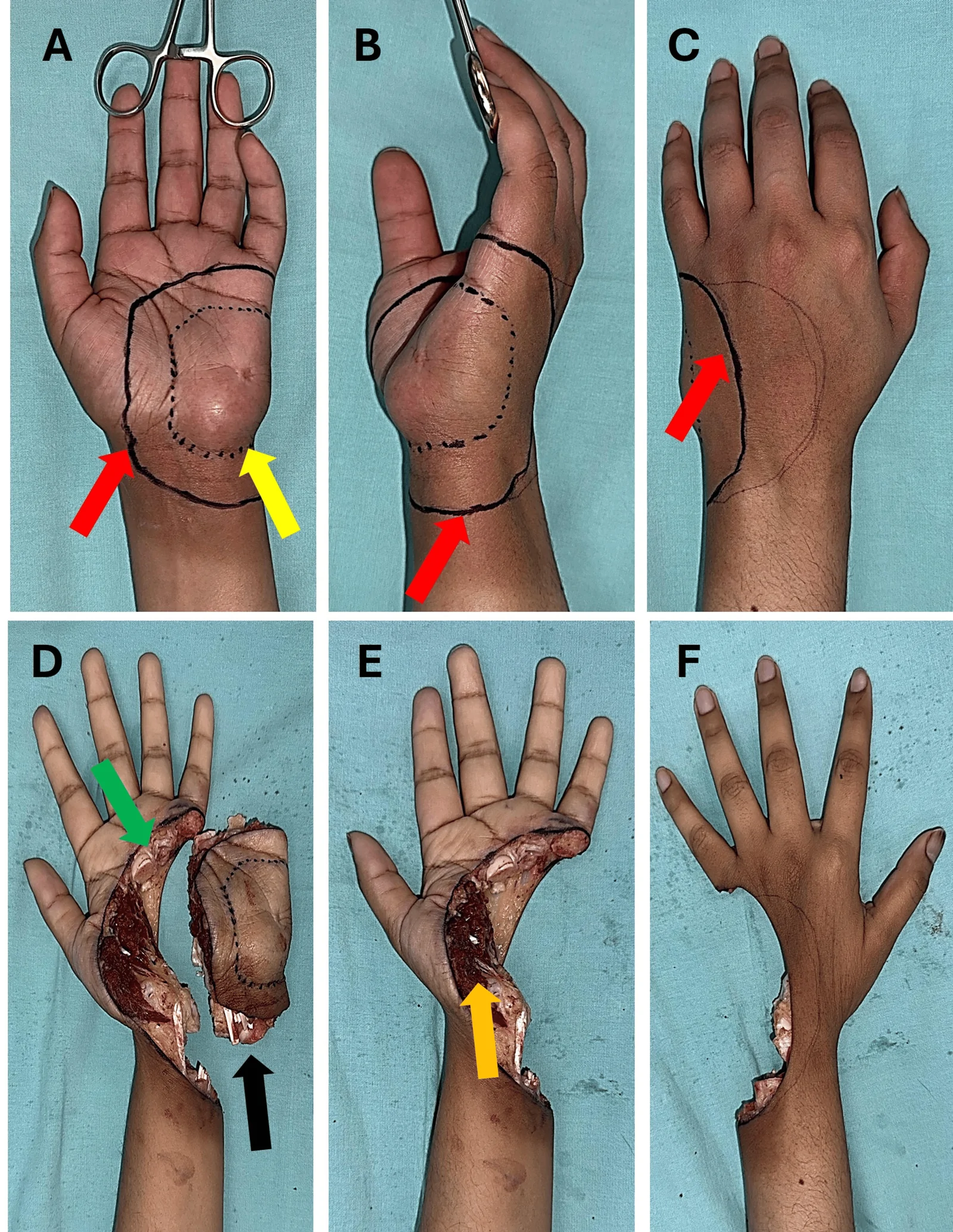
Surgical plan and design and post-intervention surgical resection. Pre-intervention A) anterior, B) lateral and C) posterior views of the marked design for resection (tumor limits, dotted line circle) with negative margins (2 cm, outer continuous line circle). Post-intervention D) anterior, E) lateral and F) posterior views of the resection performed. Red arrow: resection margin, yellow arrow: tumor margin limits, green arrow: Bone structures, Black arrow: tumor resected with margins, Orange arrow: tenar eminence muscles.

Subsequently, resection of the CCS was scheduled. Under general and regional anesthesia and tourniquet placement, marking was performed with a 2 cm (Figure 2A) margin for *en bloc* resection, including skin, diaphysis, and base of the third MTC, fourth and fifth MTC completely, and head of the ulna, as well as large, hooked, pyramidal, pisiform and lunate bone (Figure 2B, 2C) with negative margins transoperatively (Figure 2D, 2F) and preserving the neurovascular structures of the first to the fourth finger. During the same surgical time, flexor tendon reconstruction was performed with grafts: thumb flexor longus (FPL) with superficial flexor tendon (FDS), tenorrhaphy of the deep index flexor tendon with the flexor carpi ulnaris (FCU), and opposentoplasty with a tendon graft of the superficial flexor tendon of the fourth finger. Transfers from the common extensor digitorum (EDC) to the extensor digitorum are performed.

**Figure 3:**
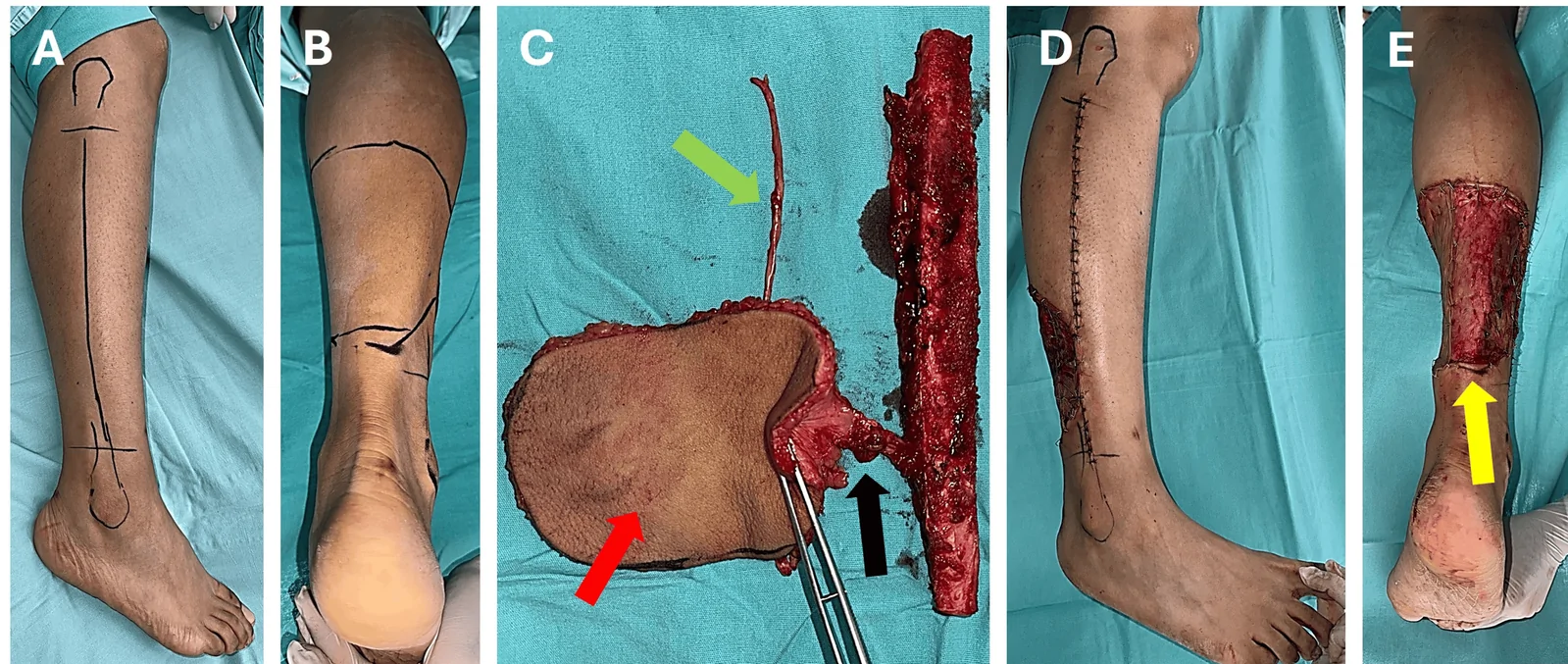
Donor site of the osteocutaneous free fibula flap. Pre-intervention marking and design of the osteocutaneous flap in the right leg in A) anterior and B) posterior view. C) Harvested free fibula flap. D) Donor site covered with partial-thickness autograft in D) anterior and E) posterior. Red arrow: cutaneous isle. Green arrow: sural nerve. Black arrow: vascular perforating pedicle. Yellow arrow: donor area covered with partial-thickness graft.

At the same time, marking was performed on the right leg for the collection of a free osteocutaneous flap (OCF) of the fibula (Figure 3A, 3B). Perforating vessels, lesser saphenous vein, sural nerve, and cutaneous island were identified (Figure 3C). The flap was harvested and the donor area was covered with a partial-thickness graft (Figure 3D, 3E).

**Figure 4:**
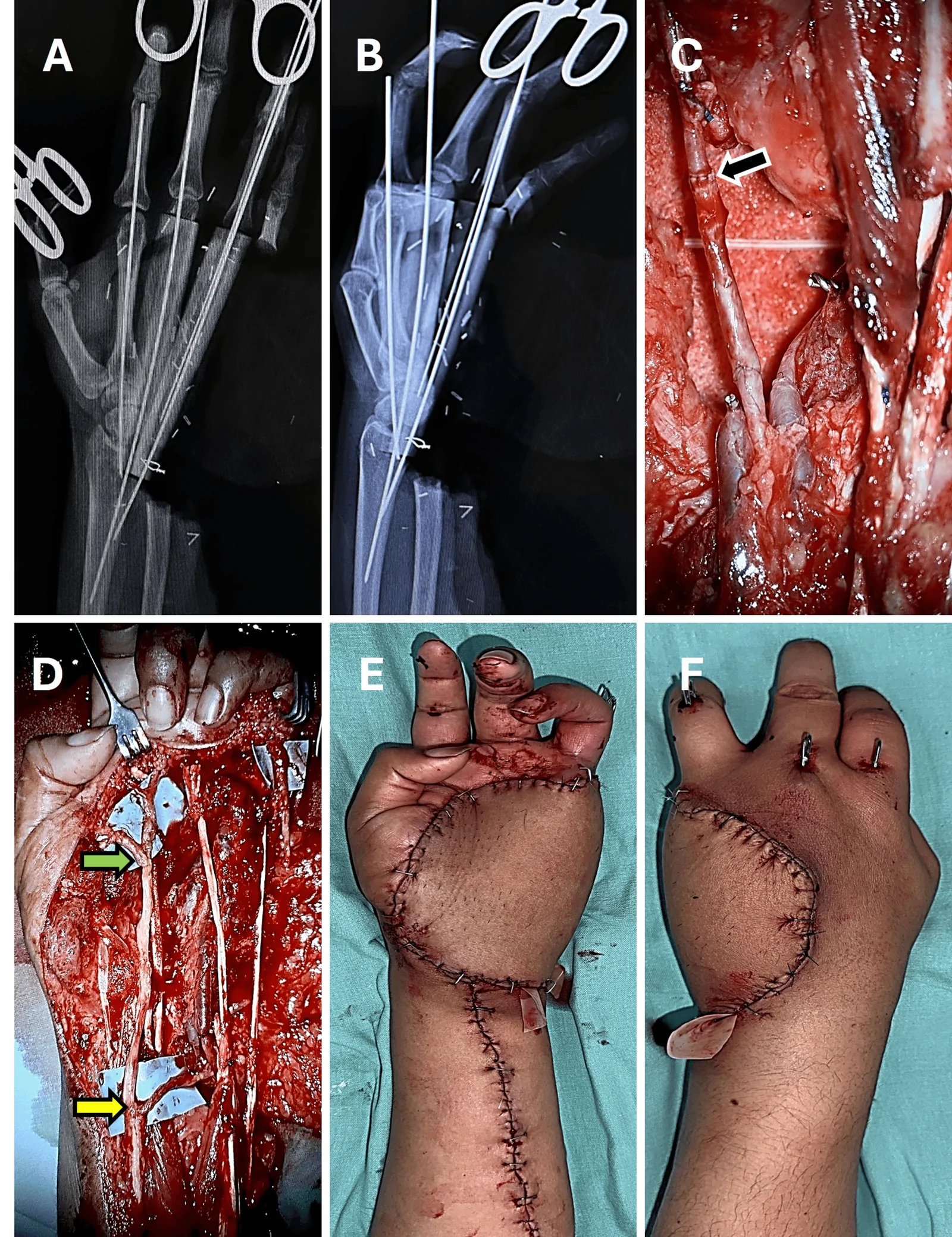
Hand reconstruction surgery after tumor resection. A) Intraoperative X-ray, showing the positioning of the 0.62 Kirschner wires in anteroposterior view and B) oblique view. C) Vascular microanastomosis, end-to-end, from the ulnar artery to the peroneal artery (black arrow). D) Intraoperative image of the hand reconstruction, showing tenorrhaphy with superficial tendon grafts to the deep flexors (green arrow) and nerve coaptations of the median nerve (yellow arrow) with an ulnar nerve graft to the digital nerves of the first, second, and third fingers and the ulnar collateral nerve of the fourth finger. E) Final postoperative image of the hand reconstruction in anterior view and F) posterior view.

At the level of the hand, ischemic changes in the fifth finger were identified and it had to be amputated within two hours of surgery; this ischemia was probably derived from the extensive tumor resection, which involved the neurovascular components of this finger. The articular facet of the radius for the lunate and head of the ulna was resected, and a retrograde intramedullary nail from the second MTC to the scaphoid and radius was inserted. The fibula was cut obliquely in the middle of its length, respecting the pedicle and a fragment of it was placed at 3° MTC towards the radius to maintain its distance and it was fixed with 0.62 Kirschner wires, performing the same procedure with the 4° MTC towards the radius and both segments of the fibula were joined in the proximal area by cerclage (Figure 4A, 4B).

Subsequently, vascular microanastomosis was performed, terminus-terminus from the ulnar artery to the peroneal artery (Figure 4C), terminus-terminal "Y" anastomosis of the concomitant fibular vein to a branch of the lesser saphenous vein, and anastomosis of the lesser saphenous vein to the cutaneous dorsal vein. The digital nerves of the median and ulnar nerves were reconstructed with sural nerve grafts, and tenorrhaphy was performed with grafting of the superficial tendons to the deep flexors and nerve coaptations (Figure 4D). Haemostasis was verified, drains were placed, and the flap was fixed with staples and sutures (Figure 4E, 4F). During his postoperative period, at approximately six hours, the flap became congested and an adjacent hematoma of approximately 50 cc was detected, which was evacuated and subsequently continued without complications. The patient was discharged after a three-day postoperative surveillance. In outpatient follow-up, a small area of the flap periphery at the wrist level suffered necrosis and was managed conservatively with closure by second intention, without complications.

The final histological report detailed a lesion of approximately 6.5 cm (diameter at the widest point, including margins), with free margins. Immunohistochemistry analysis of typical CCS markers such as metal-response element-binding transcription factor (mtf1), tumor suppressor gene p16 and transcription factor sox10 were positive. Fluorescence in situ hybridization (FISH) was positive for rearrangement of the ewsr1 gene, confirming the diagnosis.

**Figure 5:**
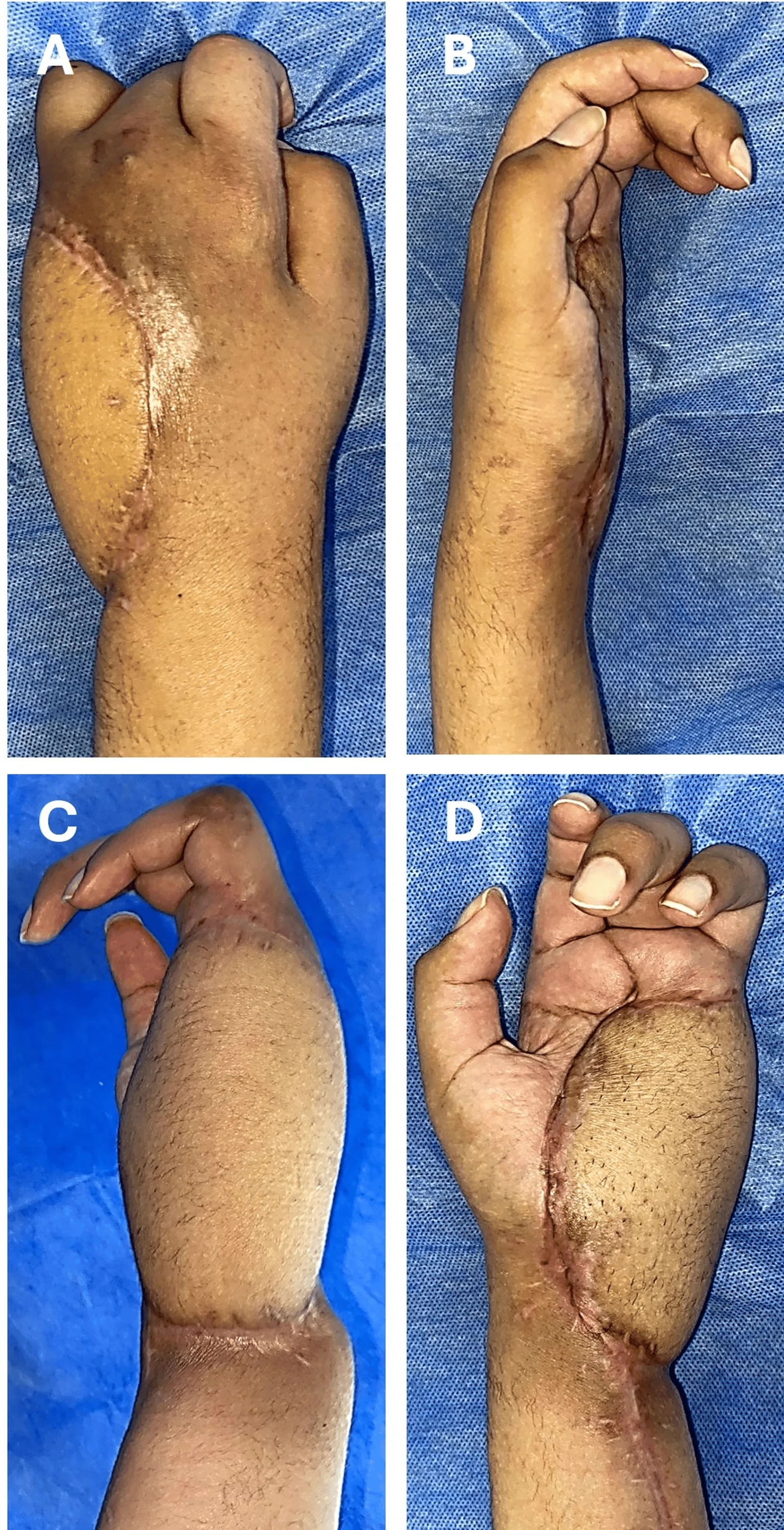
Clinical follow-up images six months after surgery. A) Dorsal view, B) Palmar (volar) view, C) radial lateral view and D) ulnar lateral view.

The patient was seen as an outpatient for six months after surgery, undergoing rehabilitation therapy (Figure 5) after a strict eight-week immobilization to optimize bone tissue union and consolidation. At that time, she had partial movement in the restored phalanges and there were no recurrences of the sarcoma. However, after this period, the hospital team was unable to maintain further follow-up or contact with her, and assessments such as Disabilities of the Arm, Shoulder and Hand (DASH) [9], Musculoskeletal Tumor Society (MSTS) [10], and grip and pressure strength could not be determined.

## Discussion

CCS is mainly located in the extremities and most frequently between the second and fourth decades of life, without sex predilection and only 10-20% are in the hand-wrist region [8]. 

Clinically, it is a slow-growing and mobile tumor; sometimes it can cause neuropathy due to nerve compression [2,8]. It tends to spread rapidly, presenting metastases at initial diagnosis in up to a third of cases [3]. Our patient's tumor had been evolving for two years and presented dimensions of 4.8 x 3.4 x 4.9 cm, without sensorineural compromise or metastasis data. A tumor size > 5 cm has been described as associated with poor survival (≤ 5 cm vs > 5 cm: 83% vs 25%) at five years [6].

Ultrasound techniques can detail a multilobulated, heterogeneous, and densely vascularized mass, suspicious of malignancy [7]. However, MRI is the diagnostic imaging method of choice; it delineates the edges of the lesion, the extent of edema, and fat involvement to support adequate surgical planning [5,11]. MRI was the reference method in our case.

For the approach, the biopsy is ideally percutaneous with an image-guided core needle, performing CT staging of the chest and abdomen [8]. In the case presented, an incisional biopsy without guidance and a chest CT scan were performed, ruling out the possibility of metastasis or adenopathies.

Preoperative radiotherapy has been reported to be useful in cases of neurovascular contact, facilitating the resection of negative margins by inducing a pseudocapsule and reducing tumor size [8]. In this sense, the reported patient achieved a significant reduction of approximately 80% in tumor size with preoperative radiotherapy. The general recommendation for adjuvant radiation therapy is made in high-grade, deep, and large (>5 cm) lesions, as in our case [3]. 

Currently, the most important and curative treatment is complete surgical resection (primary or secondary R0) with negative margins [3], which often involves amputation of the hand. Incomplete resection (R1) with positive margins has been associated with a local recurrence of 100% at two years [6].

With wide resection, mutilating surgeries such as triple central beam amputation [12] or even partial amputations of the hand are described [13]. These aggressive approaches do not improve the recurrence rate and should only be considered when the resection would lead to severe functional impairment, which could not be reconstructed, or presents considerable complications [3,8]. If lymph node involvement is suspected, dissection/biopsy should be considered, and if positive, lymphadenectomy should be considered, as it is an independent prognostic factor for recurrence and survival [8,14]. 

With tumor resection, defects composed of skin-to-bone involvement can be obtained, affecting one or more metacarpals. Therefore, a free OCF is a good reconstructive option since OCF can provide the greatest amount of bone tissue, and its intrinsic characteristics are more similar to the metacarpals that were removed [15,16]. In the case described, resection of the third, fourth and fifth MTC and a large part of the carpal bones was necessary.

The fibula-free flap has been an abutment for reconstruction of long bone defects, especially in mandibular reconstruction. The preservation of 6 cm of the fibula head can avoid complications, and it has to be limited to 4 cm [16]. Its use is ideal for metacarpal reconstruction, both for traumatic defects and for tumor resection [15], as in the case of the study.

Among the advantages of the OCF are its reliable skin pedicles, long and thin bone well vascularized with simple extraction in segments, long and good caliber vascular pedicle, a short learning curve and minimal morbidity in the donor area [15]. However, this flap is not exempt from complications such as necrosis of the cutaneous pedicle or sequelae, which, although rare, there is the possibility of graft loss in the donor area, weakness of the flexor longus hallucis and painful neuroma of the sural nerve up to gait alteration [15,16]. In our case, the cutaneous pedicle presented a small area of necrosis in its periphery, which was delimited and healed without complication.

The metastasis/recurrence risk factors include high histological grade, positive borders, 5 cm >lesion, and vascular invasion and deep plane [5,8]. The tumor treated had a dimension of ~6.5 cm diameter and the histological diagnosis of a clear cell sarcoma was confirmed with FISH (+) for rearrangement of the *ewsr*1 gene.

Follow-up MRI or CT scan is recommended every one to two years to look for recurrences or metastases, mainly to the lung (80%) and bone [5,8]. In the follow-up of our case, no abnormalities have been observed in the directed auscultation of the hand, and no recurrences were reported in six months. However, this report has limitations, especially regarding follow-up, as it was only possible to conduct it up to six months post-surgery. Furthermore, assessments using validated scales such as DASH [9], MSTS [10], or grasping abilities could not be performed due to the loss of contact with the patient. Of course, the findings and results of this report, which is based on a single-patient study, cannot be generalized to other patients with similar cases, but they could definitely help guide surgical planning and execution for limb salvage in these complex cases.

## Conclusions

Although STSs in the hand, particularly the clear cell subtype, are rare, their management requires a multidisciplinary approach. In this report, reconstruction with a fibula OCF is presented as a helpful option to restore structure and limb salvage with at least partial movement of the hand in complex defects following extensive resection.

## References

[1] Ait Addi R (2024). Metastatic clear cell sarcoma of the pancreas: an overview. World J Clin Cases.

[2] Mavrogenis AF, Panagopoulos GN, Angelini A (2017). Tumors of the hand. Eur J Orthop Surg Traumatol.

[3] Wetterwald L, Riggi N, Kyriazoglou A, Dei Tos G, Dei Tos A, Digklia A (2023). Clear cell sarcoma: state-of-the art and perspectives. Expert Rev Anticancer Ther.

[4] Enzinger FM (1965). Clear-cell sarcoma of tendons and aponeuroses. An analysis of 21 cases. Cancer.

[5] Wang WJ, Wang X, Hui DM, Feng JB, Li CM (2024). Medical imaging for the diagnosis, recurrence and metastasis evaluation of clear cell sarcoma. World J Clin Cases.

[6] Grothues J, Hardes J, Agaimy A (2024). Prognostic factors in clear cell sarcoma: an analysis of soft tissue sarcoma in 43 cases. J Cancer Res Clin Oncol.

[7] Zamora E, Zamora MA (2021). Ultrasound-based screening and characterization of an atypical clear-cell sarcoma of the hand: a case report. J Med Imaging Radiat Sci.

[8] Duran-Moreno J, Kontogeorgakos V, Koumarianou A (2019). Soft tissue sarcomas of the upper extremities: maximizing treatment opportunities and outcomes. Oncol Lett.

[9] Hudak PL, Amadio PC, Bombardier C (1996). Development of an upper extremity outcome measure: the DASH (disabilities of the arm, shoulder, and head). Am J Ind Med.

[10] Enneking WF, Dunham W, Gebhardt MC, Malawar M, Pritchard DJ (1993). A system for the functional evaluation of reconstructive procedures after surgical treatment of tumors of the musculoskeletal system. Clin Orthop Relat Res.

[11] Li Y, Huang R, Wang A, Zhang G, Su Y (2024). Is magnetic resonance imaging able to provide specific diagnoses? A case report of clear cell sarcoma of soft tissue in the first metacarpal of the hand. Case Rep Oncol.

[12] Miller SJ, Rayan GM (2000). Triple central ray amputation for clear cell sarcoma of the hand. Am J Orthop (Belle Mead NJ).

[13] Puhaindran ME, Athanasian EA (2010). Double ray amputation for tumors of the hand. Clin Orthop Relat Res.

[14] Dunaj P, Żukowska E, Czarnecka AM (2024). Lymphadenectomy in the treatment of sarcomas - indications and technique. Oncol Rev.

[15] Khan FH, Rahman O (2021). Experience of saving limbs with free fibula osteocutaneous flaps. Cureus.

[16] Addosooki A, Said E, Kenawey M, Yousef MA (2020). Reconstruction of complex hand defects using trapezoidal osteocutaneous free fibular flap. Microsurgery.

